# Species-Specific Activity of HIV-1 Vpu and Positive Selection of Tetherin Transmembrane Domain Variants

**DOI:** 10.1371/journal.ppat.1000300

**Published:** 2009-02-13

**Authors:** Matthew W. McNatt, Trinity Zang, Theodora Hatziioannou, Mackenzie Bartlett, Ismael Ben Fofana, Welkin E. Johnson, Stuart J. D. Neil, Paul D. Bieniasz

**Affiliations:** 1 Aaron Diamond AIDS Research Center and The Rockefeller University, New York, New York, United States of America; 2 Howard Hughes Medical Institute, New York, New York, United States of America; 3 Department of Microbiology and Molecular Genetics, New England Primate Research Center, Harvard Medical School, Southborough, Massachusetts, United States of America; Northwestern University, United States of America

## Abstract

Tetherin/BST-2/CD317 is a recently identified antiviral protein that blocks the release of nascent retrovirus, and other virus, particles from infected cells. An HIV-1 accessory protein, Vpu, acts as an antagonist of tetherin. Here, we show that positive selection is evident in primate tetherin sequences and that HIV-1 Vpu appears to have specifically adapted to antagonize variants of tetherin found in humans and chimpanzees. Tetherin variants found in rhesus macaques (rh), African green monkeys (agm) and mice were able to inhibit HIV-1 particle release, but were resistant to antagonism by HIV-1 Vpu. Notably, reciprocal exchange of transmembrane domains between human and monkey tetherins conferred sensitivity and resistance to Vpu, identifying this protein domain as a critical determinant of Vpu function. Indeed, differences between hu-tetherin and rh-tetherin at several positions in the transmembrane domain affected sensitivity to antagonism by Vpu. Two alterations in the hu-tetherin transmembrane domain, that correspond to differences found in rh- and agm-tetherin proteins, were sufficient to render hu-tetherin completely resistant to HIV-1 Vpu. Interestingly, transmembrane and cytoplasmic domain sequences in primate tetherins exhibit variation at numerous codons that is likely the result of positive selection, and some of these changes coincide with determinants of HIV-1 Vpu sensitivity. Overall, these data indicate that tetherin could impose a barrier to viral zoonosis as a consequence of positive selection that has been driven by ancient viral antagonists, and that the HIV-1 Vpu protein has specialized to target the transmembrane domains found in human/chimpanzee tetherin proteins.

## Author Summary

Tetherin is a cell surface protein that acts as an antiviral defense. It functions by tethering newly assembled HIV-1 particles to the surface of the infected cell, such that the viral particle is unable to depart and disseminate to other, uninfected cells. HIV-1 possesses an antagonist of tetherin, termed Vpu, that abolishes tetherin function. We found that HIV-1 is an effective antagonist of human and chimpanzee variants of tetherin but is unable to antagonize tetherins from two monkey species. Additionally, we found that sequence differences in a portion of the protein that is embedded in cell membranes determined whether or not it could be antagonized by Vpu. Since the Vpu protein is alsi a membrane embedded protein, this result suggests that Vpu and tetherin interact within cell membranes. We also show that tetherin has been evolving rapidly, and has likely been placed under selective pressure to change sequence. Notably, portions of tetherin that appear to have been placed under selective pressure coincide with positions that influence Vpu antagonism. Therefore, the evolutionary history of primates determines the effectiveness of HIV-1 Vpu in modern species. Thus, tetherin could impose a barrier to cross species transmission of retroviruses.

## Introduction

Eukaryotic cells can constitutively or inducibly express a variety of molecules that inhibit the replication of viruses. Among these antiviral defenses are components of the type-I interferon (IFN) -induced innate immune system [1],[2]. In turn, viruses have evolved to express proteins that either limit IFN-induced gene expression or directly antagonize the function of antiviral proteins.

We and others recently identified an IFN-induced antiviral protein, termed tetherin, that functions by a novel mechanism. Specifically, tetherin blocks the release of nascent virions from HIV-1 infected cells [3]–[5]. Tetherin is an integral membrane protein with a unique topology. In particular, it encodes a transmembrane anchor towards its N-terminus, as well as a putative glycophosphatidyl-inositol lipid anchor at its C-terminus [6]. These two membrane anchors are linked by an extracellular domain that is predicted to form a coiled-coil. Ectopic expression of tetherin in cells that do not ordinarily express it results in the formation of protease-sensitive tethers that causes retention of retrovirus particles on the surface of infected cells, from where they can be internalized [4],[5],[7],[8]. This pronounced ability to retain and internalize HIV-1 particles is present constitutively in cells that normally express tetherin, but is suppressed when tetherin is depleted. Tetherin colocalizes with Gag and appears to act by inducing adherence of virion and cell membranes. Thus, virions that are retained by tetherin are fully formed and mature, and have lipid bilayers that are discontinuous with cell membranes [4],[7].

Notably, an HIV-1 accessory transmembrane protein, Vpu, acts as a viral antagonist of tetherin [4],[5]. Indeed tetherin dramatically inhibits the release of Vpu-defective HIV-1 virions, but has only modest effects on wild-type Vpu-expressing HIV-1. Moreover, Vpu colocalizes with tetherin and prevents the localization of tetherin to nascent virions, perhaps through its ability to reduce the amount of tetherin at the cell surface [4],[5]. Thus, the existence of tetherin explains the previously observed requirement for Vpu during HIV-1 particle release from certain cells, particularly those that have been exposed to type-I IFN [3], [7], [9]–[12].

The wide expression of tetherin upon exposure of cells to IFN-alpha [4],[13] and the wide range of retroviruses and filoviruses that are inhibited by tetherin [8] suggests that it might be a general component of an innate immune defense against many enveloped viruses. As such, tetherin could provide an impetus for the evolution of antagonists in viruses other than HIV-1. Indeed, the Kaposi's sarcoma herpesvirus (KSHV) also encodes a likely antagonist of tetherin, since expression of the KSHV K5 protein decreases the steady state level of tetherin protein [14]. Additionally, certain retroviral envelope proteins, in particular the HIV-2 Env, have Vpu-like activity [15],[16]. Thus, it seems likely that tetherin antagonists other than Vpu exist.

Here, we show that tetherin proteins from different species exhibit marked differences in sensitivity to antagonism by HIV-1 Vpu. Specifically, tetherin proteins from two old world monkey species as well as from mouse, are effective inhibitors of HIV-1 particle release, but are resistant to Vpu. Moreover, we show that the transmembrane domain of tetherin contains determinants of sensitivity to Vpu, and that two mutations in the human tetherin sequence are sufficient to generate a protein that is entirely resistant to antagonism by Vpu. Interestingly, tetherin sequences that are predicted to be accessible to cytoplasmic or integral membrane antagonists, such as HIV-1 Vpu, exhibit evidence of positive selection at numerous codons, including some that we demonstrate determine the effectiveness of Vpu antagonism. Thus, past selective pressures imposed on tetherin by viral antagonists likely provides a barrier to the establishment of zoonotic infections by modern primate lentiviruses, and potentially enveloped viruses from other species, whose spread depends on antagonism of tetherin function.

## Results

### Resistance of nonhuman tetherin proteins to antagonism by HIV-1 Vpu

**Figure 1:**
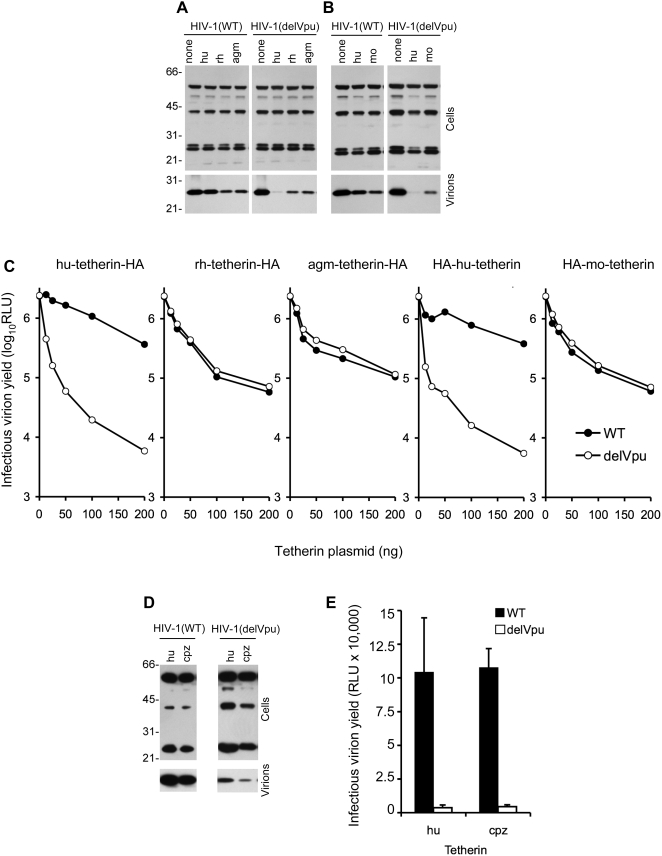
Effect of nonhuman tetherins on HIV-1 particle release and antagonism by Vpu. A, B) Western blot analysis (anti HIV-1 capsid, p24) of cell and virion lysates following transfection of cells with WT and Vpu deleted (delVpu) proviral plasmids, alone (none) or in combination with 50 ng of the indicated human (hu) rhesus monkey (rh) African green monkey (agm) tetherin-HA expression plasmids (A) or 50 ng of hu- or mouse (mo) HA-tetherin expression plasmids (B). Numbers to the left of the blots indicate the positions of molecular weight markers. C) Infectious virion yield, measured using HeLa-TZM indicator cells and given in relative light units (RLU), following transfection of cells with WT and Vpu deleted (delVpu) proviral plasmids, in combination with varying amounts of the indicated tetherin-HA or HA-tetherin expression plasmids. D) Western blot analysis (anti HIV-1 capsid, p24) of cell and virion lysates following transfection of cells with WT and Vpu deleted (delVpu) proviral plasmids in combination with 100 ng of untagged human (hu) or chimpanzee (cpz) tetherin expression plasmids. Numbers to the left of the blots indicate the positions of molecular weight markers. E) Infectious virion yield, measured using HeLa-TZM indicator cells and given in relative light units (RLU), following transfection of cells with WT and Vpu deleted (delVpu) proviral plasmids, in combination with 100 ng of hu-tetherin or cpz-tetherin expression plasmids.

Inspection of sequence databases revealed the presence of putative tetherin proteins in various mammalian species. For functional analyses, we amplified tetherin sequences from cDNAs derived from rhesus macaque (rh), African green monkey (agm), chimpanzee (cpz) and mouse (mo). Coexpression of hu-tetherin, cpz-tetherin, rh-tetherin, agm-tetherin, mo-tetherin, with HIV-1 (delVpu) caused a marked decease in the yield of viral particles, as measured using infectivity or western blot assays (Fig. 1 A,B,C). The magnitude of the reduction in virus yield varied somewhat, depending which tetherin protein was coexpressed. In each case, inhibition of virion release by tetherin did not lead to a dramatic accumulation of cell associated viral proteins. This finding suggest that the virions that are retained by tetherin, are destroyed at rate that exceeds, or is not greatly different, to their synthesis. This would likely be through endocytosis, followed by lysosomal degradation. Moreover, it may simply be the case that only a fraction of the viral protein that is synthesized by infected cells is actually released as particles. Thus the amount of viral protein that is observed in cell lysates would be determined by its intrinsic turnover rate, rather than particle release versus retention.

The differences in potency that were observed among the various tetherin proteins were only partly explained by variation in the levels at which each tetherin was expressed (Fig. 1, Fig. S1A), A finding which suggests that natural variation in potency may exist among mammalian tetherin proteins, and that hu-tetherin is particularly potent. However, a caveat to be attached to this conclusion is that transiently expressed tetherin exisits as a variety of species, presumably reflecting heterogeneous glycosylation. At present it is not clear whether all of the various tetherin species are active, and it is possible that the amount of active tetherin is not uniformly represented in analyses of total tetherin protein levels. Nonetheless, each tetherin protein was clearly capable of reducing infectious HIV-1(delVpu) yield, by 20-fold or more. Strikingly, however, and in contrast to hu-tetherin, the rh-, agm- and mo-tetherin proteins were equally efficient in reducing HIV-1(WT) and HIV-1(delVpu) virion yield (Fig. 1A,B,C). Conversely, as with hu-tetherin, HIV-1(WT) was substantially resistant to inhibition by cpz-tetherin, while the release of HIV-1(delVpu) particles was strongly inhbited (Fig. 1 D, E). Thus, the non-hominid tetherin proteins were apparently insensitive to antagonism by HIV-1 Vpu. while both hu-tetherin and cpz-tetherin were Vpu-sensitive.

### Tetherin transmembrane sequences can transfer resistance or sensitivity to Vpu

**Figure 2:**
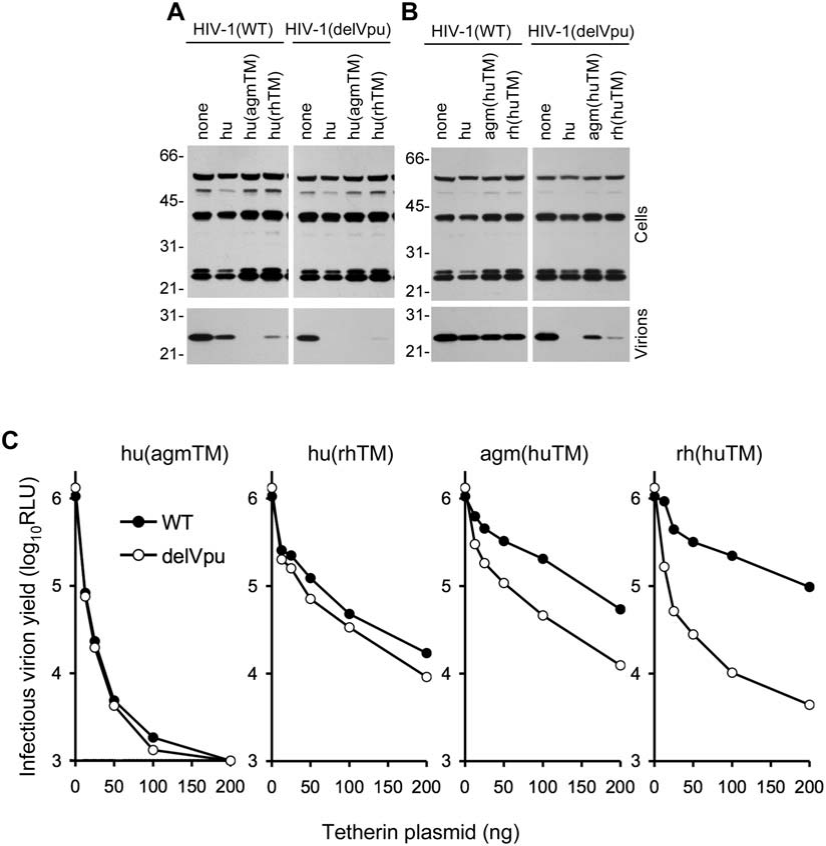
Tetherin TM domains are determinants of sensitivity/resistance to HIV-1 Vpu. A, B) Western blot analysis (anti HIV-1 capsid, p24) of cell and virion lysates following transfection of cells with WT and Vpu deleted (delVpu) proviral plasmids, either alone (none) or in combination with 50 ng of plasmids expressing chimeric human tetherin-HA proteins encoding monkey-derived TM domains (A), or monkey tetherin-HA proteins encoding a human tetherin TM domain (B). C) Infectious virion yield, measured as in Fig. 1, following transfection of cells with WT and delVpu proviral plasmids, with varying amounts of the indicated tetherin-HA expression plasmids.

Earlier work indicated that expression of an intact transmembrane (TM) segment of HIV-1 Vpu was most important, and perhaps sufficient, to enhance HIV-1 particle release [17]. This finding, combined with the notion that only the N-terminal portion of tetherin should be physically accessible to Vpu, suggested the possibility that Vpu might target the transmembrane domain of tetherin. Thus, we generated chimeric proteins, termed hu(agmTM) and hu(rhTM), in which the transmembrane domain of hu-tetherin was replaced with corresponding sequences from rh- or agm-tetherin. These chimeric proteins differed in the magnitude with which they inhibited HIV-1 virion release, concordant with differences in expression level (Fig. S1B), but both were entirely resistant to antagonism by Vpu (Fig. 2A, C). Indeed, the hu-tetherin protein containing the agm TM domain inhibited both HIV-1(delVpu) and HIV-1(WT) particle release with similar or greater potency with which the intact hu-tetherin protein selectively blocked HIV-1(delVpu) release (Fig 2A, C).

In a reciprocal experiment, rh-tetherin and agm-tetherin proteins encoding a hu-tetherin transmembrane domain were generated. The rh(huTM) protein was expressed at a slightly higher level and was a more effective inhibitor of HIV-1 particle release than was the agm(huTM) protein (Fig. 2B,C, Fig. S1B). However, both proteins selectively inhibited HIV-1(delVpu) particle release, indicating that they were sensitive to antagonism by HIV-1 Vpu. Thus, the exchange of the TM domain between tetherin proteins from different species transferred sensitivity and resistance to antagonism by HIV-1 Vpu.

### Determinants of Vpu sensitivity in human tetherin

**Figure 3:**
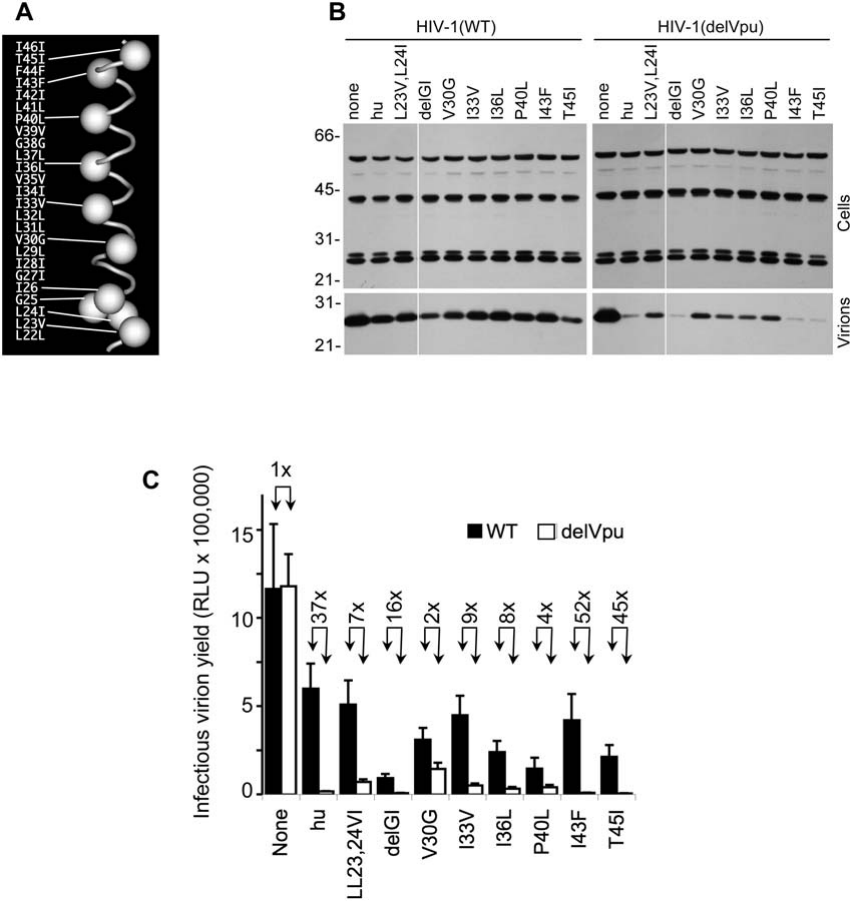
Effects of individual TM domain mutations on tetherin potency and Vpu antagonism. A) Differences between hu- and rh-tetherin TM domains. The human (left) and rhesus (right) sequences are depicted, from the cytoplasmic face (lower) to the extracellular face (upper) of the plasma membrane; an integrin beta3 transmembrane segment was used to model the position of the differences. B) Western blot analysis (anti HIV-1 capsid, p24) of cell and virion lysates following transfection of cells with WT and Vpu deleted (delVpu) proviral plasmids, alone (none) or in combination with 50 ng of the indicated unmanipulated or mutant hu-tetherin-HA expression plasmids. C) Infectious virion yield, measured as in Fig. 1, following transfection of cells with WT and Vpu deleted (delVpu) proviral plasmids, in combination with 50 ng of the indicated mutant hu-tetherin-HA expression plasmids. Numbers above each pair of bars indicate the fold difference in virion yield when WT and delVpu viruses are compared.

**Figure 4:**
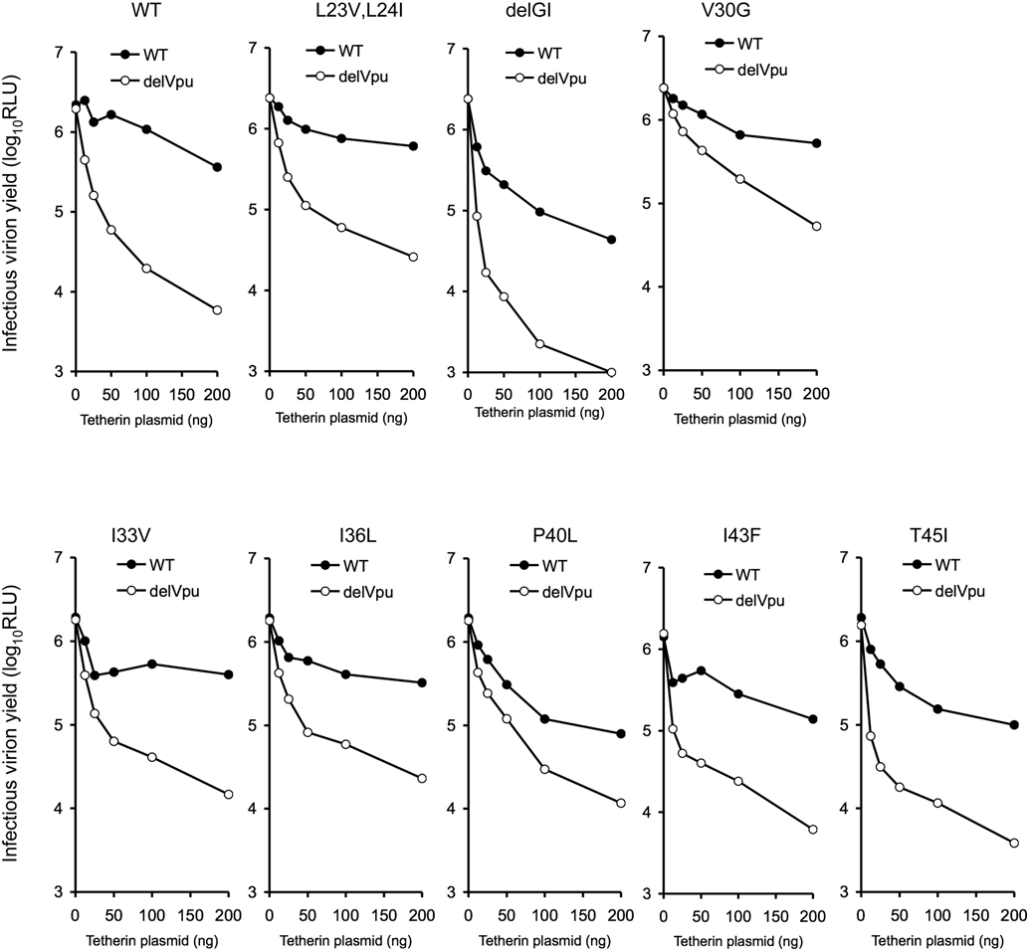
Effects of individual TM domain mutations on tetherin potency and Vpu antagonism. Each chart shows infectious virion yield, measured as in Fig. 1, following transfection of cells with WT and Vpu deleted (delVpu) proviral plasmids, in combination with varying amounts of the indicated mutant hu-tetherin-HA expression plasmids.

Inspection of tetherin TM domain protein sequences revealed several differences between the monkey and human proteins, distributed along the length of the TM domain (Fig. 3A). To determine which of these were responsible for the Vpu resistance of the monkey tetherin proteins to antagonism by Vpu, we generated mutant forms of hu-tetherin, each bearing an individual change in the TM domain that is found at the corresponding position in rh-tetherin. (The exceptions to this scheme were the L23V,L24I mutant in which two contiguous amino acids were changed to their rh-tetherin counterparts and the delGI change, where a two amino acid deletion that is present in the rh-tetherin sequence was introduced). This panel of mutant tetherin proteins varied in the potency with which they reduced HIV-1(delVpu) virion yield, and in their ability to be antagonized by Vpu (Fig. 3B,C). Variation in the potency of antiviral activity among the mutant panel correlated with expression level in most cases (Fig. S1C). Notably, none of the individual hu-tetherin mutants recapitulated the phenotype of the rh-tetherin or hu(rhTM)-tetherin proteins that appeared completely resistant to Vpu antagonism. Rather, several of the individual mutants appeared partly resistant to antagonism by Vpu, in that Vpu was not able enhance virion release as effectively in their presence as it did in the presence of unmanipulated hu-tetherin (Fig. 3B,C). Because these analyses were slightly confounded by the variation in the potency and expression of the individual tetherin mutants, we measured infectious HIV-1(WT) and HIV-1(delVpu) virion yield in the presence of varying levels of the mutant tetherin proteins (Fig. 4). Overall, these analyses identified a single amino acid difference (P40L) as contributing substantially to the Vpu sensitivity of hu-tetherin and the Vpu-resistance of rh-tetherin. Other changes in the hu-tetherin TM had more modest effects, or no effect on Vpu sensitivity, when present in isolation, or substantially affected tetherin potency (Fig. 3, Fig. 4).

**Figure 5:**
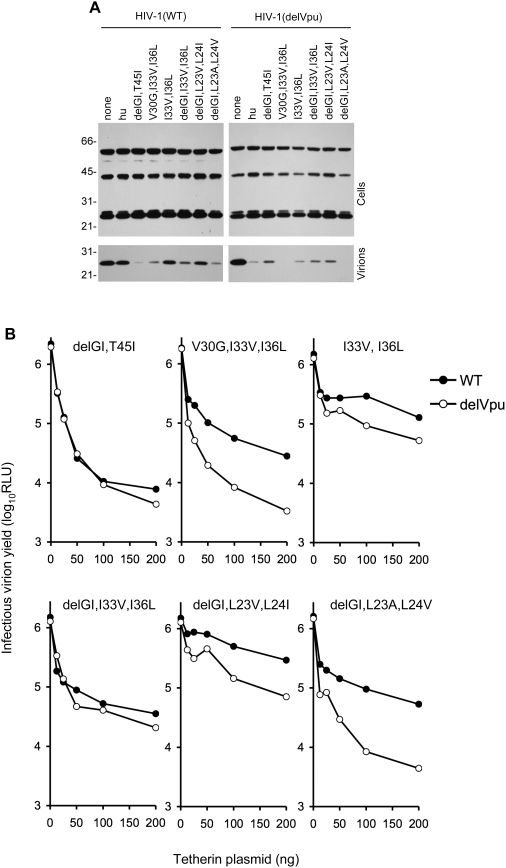
Effects of combined TM domain mutations on tetherin potency and Vpu antagonism. A) Western blot analysis (anti HIV-1 capsid, p24) of cell and virion lysates following transfection of cells with WT and Vpu deleted (delVpu) proviral plasmids, alone (none) or in combination with 50 ng of the indicated unmanipulated or mutant hu-tetherin-HA expression plasmids. B) Infectious virion yield, measured as in Fig. 1, following transfection of cells with WT and delVpu proviral plasmids, with varying amounts of the indicated mutant tetherin-HA expression plasmids.

Because our initial analysis revealed that no single change in the TM domain of hu-tetherin could abolish sensitivity to Vpu, we next tested whether mutations that individually had minor or partial effects on Vpu antagonism could exert more dramatic effects when present in combination. In particular, several mutations were combined with the most obvious difference between human and monkey tetherin TM domains, namely the delGI change. Notably, the delGI,T45I double mutant strongly inhibited HIV-1 particle release and was completely resistant to antagonism by Vpu (Fig. 5A,B). Additionally, the I33V,I36L combination mutation which would be predicted to target proximal residues on the face of a TM alpha helix (Fig. 3A) appeared to confer at least partial resistance to antagonism by Vpu. However, this double mutation generated a tetherin protein with only modest activity. Nonetheless, when combined with a mutation at a third proximal residue (generating V30G,I33V,I36L) this combined mutation conferred partial resistance to Vpu antagonism, in the context of a protein with potent antiviral activity (Fig. 5A, B). Moreover a hu-tetherin bearing combined delGI,I33V,I36V mutations was almost completely resistant to antagonism by Vpu, and exhibited substantial antiviral activity (Fig. 5A, B). Finally, combining the delGI mutation with subsitiutions at contiguous residues that corresponded to rh-tetherin residues (delGI, L23V,L24I) resulted in a protein with only weak inhibitory activity, whereas combining the delGI mutation with contiguous L23A,L24V mutations (corresponding to agm-tetherin residues) generated a protein that was potent and partly Vpu resistant (Fig. 5A, B). Notably, the differences in activity and Vpu sensitivity among the various combination-mutant tetherin proteins was not explained by differences in expression level (Fig. S1D).

**Figure 6:**
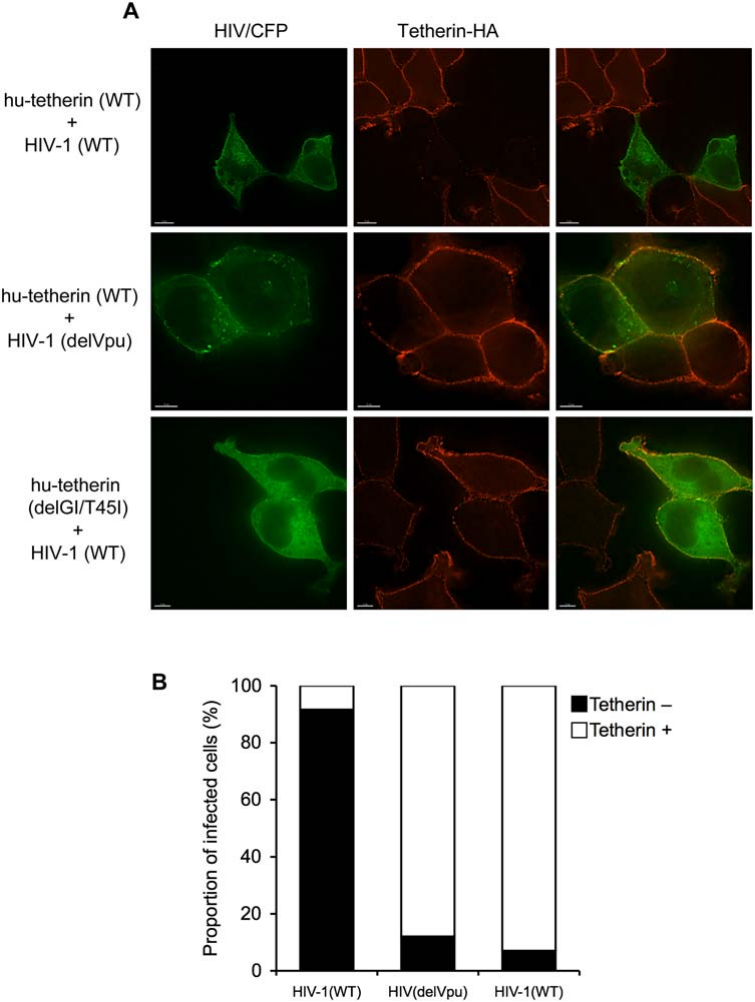
A tetherin mutant that is resistant to antagonism by Vpu is also resistant to surface downregulation by Vpu. A) 293T cells stably expressing either WT or mutant (delGI/T45I) hu-tetherin were infected with VSV-G pseudotyped HIV-1(WT) or HIV-1(delVpu) variants that carried Cerulean-FP (CFP, green), as indicated. Cells were fixed but not permeabilized in order to confine tetherin-HA staining to surface expressed protein (red). B) Cells were scored visually for the presence (tetherin +) or absence (tetherin −) of intense tetherin-HA staining on the cell's surface (see examples in panel (A)). Greater than 95% of the uninfected WT or delGI/T45I hu-tetherin-HA expressing cells exhibited intense surface tetherin-HA expression. At least seventy individual CFP-positive infected cells were evaluated for surface tetherin-HA expression each experimental condition.

To determine whether mutations that conferred resistance to antagonism by Vpu in virion release assays also conferred resistance to the previously described phenomenon of Vpu-induced downregulation of hu-tetherin from the cell surface [5], we generated cell lines stably expressing either wild type hu-tetherin-HA protein or the Vpu-resistant delGI/T45I hu-tetherin mutant (Fig. 6). Upon infection with HIV-1 (WT), hu-tetherin was efficiently depleted from the surface of the vast majority of infected cells (Fig. 6A, B). Conversely, infection with HIV-1 (delVpu) resulted in little or no hu-tetherin downregulation from the surface of infected cells. Strikingly, and unlike the WT hu-tetherin protein, the delGI/T45I mutant hu-tetherin was not removed from the cell surface upon infection with HIV-1 (WT) (Fig. 6A, B) and, thus, was resistant to surface downregulation by Vpu.

Overall, these experiments revealed that no single difference between the hu-tetherin and rh-tetherin proteins accounted for their respective sensitivity and resistance to antagonism by HIV-1 Vpu. Rather, they indicated that the particular combination of residues in the tetherin TM domain can affect antiviral potency, and that multiple differences between human and monkey proteins, including the delGI indel, and the I33V, I36L, P40L and T45I differences, influence the differential sensitivity of tetherins to antagonism by Vpu.

### Intraspecies polymorphism and evidence of positive selection in tetherin

**Figure 7:**
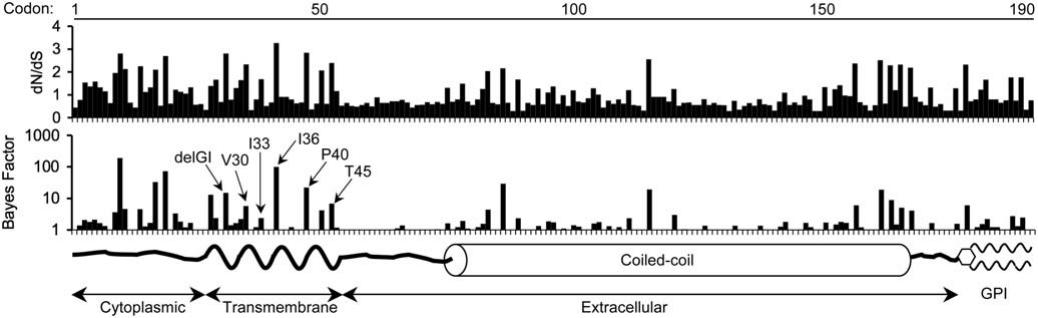
Positive selection in primate tetherin sequences. A diagram representing the various domains of the tetherin protein is shown, below plots of the dN/dS ratio (upper plot) and the Bayes factor for dN>dS (lower plot) at each codon in an alignment of primate tetherin coding sequences. Sequences from human, chimpanzee, gorilla, gibbon, African green monkey, pigtail macaque (2 variants), rhesus macaque (4 variants), long tail macaque (2 variants), sooty mangabey (2 variants), and marmoset were used in the analysis (see Fig. S2 for the codon alignment). Also indicated are residues in hu-tetherin that contribute to HIV-1 Vpu sensitivity.

Inspection of a larger collection of mammalian tetherin sequences amplified from various old world primates, or retrieved from sequence databases, revealed some striking features. Firstly, among the nonprimate mammalian tetherin sequences, the N-terminal cytoplasmic domain was hypervariable, both in length and sequence (data not shown). Because of these properties, it proved impossible to unambiguously align non-primate and primate tetherin sequences in order to perform tests for positive selection. Therefore, we confined further analyses to primate tetherin sequences, which could be aligned unambiguously throughout the entire length of the coding sequence. Even within primates there was considerable sequence divergence between species, ranging from 0.5% to 40.0% at the nucleotide level. Sampling within three old world monkey species (rhesus macaques, pig-tailed macaques and sooty mangabeys) also revealed the presence of significant polymorphism within species (Fig. S2 and data not shown); it remains to be seen whether nonsynonymous polymorphism extends to the *tetherin* locus of other primate lineages. Positive selection was tested using the REL(HyPhy) [18] and CODEML (PAML) [19] methods and these analyses revealed that codons exhibiting high dN/dS ratios, and therefore likely to have been subjected to positive selection, were enriched in the N-terminal cytoplasmic and TM domains in primate tetherins (Fig. 7). Tetherin evolution in primates was also evaluated under several standard models of sequence evolution as implemented in the CODEML program. These comprise three nested pairs of models (M0 and M3; M1a and M2a; M7 and M8) in which the second model of each pair is derived from the first by allowing sites to evolve under positive selection. Nested models were compared using the likelihood ratio test, and in each case allowing individual sites to evolve under positive selection (M3, M2a, M8) gave a significantly better fit to the primate sequence data than the corresponding model without positive selection (M0, M1a and M7, respectively) (Table 1). The M3, M2a and M8 models identified a largely overlapping set of sites in the tetherin coding sequence with dN/dS>1, consistent with an evolutionary history characterized by frequent episodes of positive selection. Notably, some codons that exhibited a high probability of having evolved under positive selection coincided with residues that determined the effectiveness of Vpu antagonism (Fig. 7). However, there were numerous additional codons, particularly in the tetherin cytoplasmic domain, that also exhibited high dN/dS ratios, suggesting that antagonists other than Vpu have also imposed selective pressure on primate tetherin sequences.

**Table 1:** Models of evolution applied to primate tetherin sequences.

Comparison	2(Ln_1_-Ln_0_)*	d.f.	χ^2^
M0, M3	40.8	4	p<0.00001
M1a, M2a	14.2	2	p<0.0008
M7, M8	13.8	2	P<0.001

*: The likelihood ratio test (LRT) was used to compare two nested models of sequence evolution, in which one model is a more complex version of the other. The LRT is used to justify the additional assumptions that are incorporated in the more complex model (or to reject the more complex model in favor of the simpler model) by asking whether the additional assumptions result in a statistically significant improvement in fit to the data. For the three comparisons indicated, the second model only differs from the first in permitting some form of positive selection to act on individual sites. The LRT is conducted by calculating twice the difference in maximum likelihood scores for the two models under comparison and then finding the chi-square critical value. For a recent discussion of these models and their evaluation using the LRT, see reference [24].

d.f. - degrees of freedom.

## Discussion

Previously, we reported that Vpu could reverse the inhibitory effect of IFN-alpha on HIV-1 particle release from human cells, but that Vpu failed to reverse such IFN-alpha induced inhibition in African green monkey cells [3]. The subsequent discovery that tetherin is an IFN-induced inhibitor that is antagonized by Vpu [4],[5] leads to the prediction that agm-tetherin should be resistant to Vpu. Here we show that agm-tetherin, as well as rh-tetherin and mo-tetherin are indeed resistant to antagonism by Vpu, in contrast to tetherin variants found in species (human and chimpanzee) that are permissive hosts for HIV-1. Moreover, these studies identify the TM domain of hu-tetherin as a major determinant of the effectiveness of Vpu antagonism. That the TM domain of tetherin harbors critical determinants of Vpu sensitivity is concordant with previous observations indicating that the TM domain of Vpu is critical for its virus release activity [17]. Thus, these observations suggest a model in which Vpu and tetherin interact via their TM domains. Such an interaction could be direct, but further work will be required to resolve whether this is indeed the case, and precisely how Vpu tetherin antagonism and/or downregulation from the cell surface is achieved is not known. In this regard, the recent report suggests that the host cell protein CAML is important for Vpu activity [20], but how tetherin, Vpu and CAML interact in a functional sense is unclear at present.

Importantly, no single change in the hu-tetherin TM domain abolished Vpu sensitivity, which is consistent with a model in which Vpu, or a bridging factor, makes multiple contacts with the TM domain of tetherin. Indeed, the hu-tetherin mutant, delGI,T45I, that had the most striking phenotype, in that it retained full activity but was completely resistant to antagonism by Vpu, harbored mutations at positions close to the opposing ends of the TM domain. Additionally, a different combination of mutations (delGI, I33V, I36L) also conferred complete Vpu resistance, again consistent with the notion that multiple contacts with the tetherin TM domain are made during its antagonism by Vpu. Concordant with these findings, the chimpanzee and human tetherin proteins were both sensitive to HIV-1 Vpu, and differed from each other at only a single position in their TM domains. Thus, it is likely only minor, if any, adaptation in the SIV_CPZ_ Vpu protein that is immediately ancestral to the HIV-1 Vpu proteins would have been required in order for it to target human tetherin.

Primate tetherin TM domain sequences, that should only be accessible to integral membrane antagonists, exhibit clear evidence of positive selection at several codons, including some that determine the effectiveness of Vpu antagonism. Thus, it is likely that antagonists encoded by pathogenic viruses have driven the selection of the tetherin variants that exist in modern primates. Such antagonists could include Vpu itself, since several primate lentiviruses encode Vpu proteins (http://www.hiv.lanl.gov/). However, other viral antagonists, including the KSHV K5 protein [14], or homolgues of it, are also reasonable candidates for factors that have imposed selective pressure on tetherin sequences. In addition to the TM, several sites with the highest dN/dS ratios mapped to the N-terminal cytoplasmic domain. Such sites may define an accessible target exploited by other virally encoded inhibitors of tetherin. It is noteworthy in this regard that many primate lentiviruses do not encode a Vpu like protein, and may have evolved alternative strategies targeting this or other regions of the protein. Additionally, tetherin sequences might also have evolved to better target specific types of viral particles in some host species, as a consequence of varying viral challenges. Such evolution might also include adaptations in domains of tetherin that, for example, modify its trafficking within cells (likely including the cytoplasmic and TM domains). Overall, there are several potential sources of evolutionary pressure that could give rise to positive selection and diversification of tetherin genes. We note that the sequence of the TM domain of tetherin is obviously constrained by the need to retain the biochemical characteristics of a TM domain, which might mitigate against the detection of positive selection in this protein domain.

HIV-1 is well adapted to replicate in human cells, but fails to replicate in many nonhuman primate cells. This is in large part because it is unable to evade or antagonize the species-specific variants of antiviral genes, such as TRIM5 and APOBEC3, which show evidence of positive selection that is assumed to have resulted from past retroviral epidemics [21]–[23]. Tetherin represents a third example of an antiviral gene in monkeys that exhibits activity against intact HIV-1 as a consequence of positive selection in the primate lineage. Thus an array of antiviral molecules limit the replication of primate lentiviruses in non-natural host cells, creating barriers to zoonosis, and revealing potential opportunities to mobilize intrinsic antiretroviral defenses by therapeutic inhibition of the activity of their viral antagonists.

## Materials and Methods

### Plasmid construction

A hu-tetherin cDNA, cloned into pCR3.1 vector, and its N-terminally HA tagged counterpart have been described previously [4]. An internally HA-tagged tetherin expression construct, pCR3.1/hu-tetherin-HA was derived from this by inserting an NheI restriction site at nucleotide position 463 of the tetherin gene. Thereafter, complimentary oligonucleotides encoding an HA epitope tag were inserted into the NheI site. Similarly, the tetherin coding sequence from rhesus macaque, African green monkey chimpanzee and mouse was amplified using cDNA generated from IFN-alpha treated 221, COS-7, chimpanzee fibroblasts, and NIH3T3 cells respectively. For the monkey tetherins, the HA epitope was inserted at a position orthologous to nucleotide 463 as described above, while a N-terminally tagged murine tetherin construct was used. Thereafter, overlap-extension PCR approaches were used to exchange TM domain segments between human and monkey proteins, or to introduce point mutations into the hu-tetherin sequence.

### Cells and transfection

293T cells were maintained in DMEM media supplemented with 10% fetal calf serum and gentamycin, as were HeLa-TZM cells which express CD4 and CCR5 and contain a *lacZ* reporter gene under the control of an HIV-1 LTR. To measure tetherin and Vpu activity, 293T cells were seeded in a 24 well plate at a concentration of 1.5×10^5^ cells/well and transfected the following day using polyethylenimine (PolySciences) with 500 ng of an unmanipulated HIV-1 proviral plasmid NL4-3(WT) or a Vpu-defective counterpart NL4-3(delVpu). Additionally, 50 ng of a tetherin expression plasmid and 50 ng pCR3.1/cherry fluorescent protein (to monitor transfection efficiency) were included in the transfection. In experiments where the level of tetherin was varied, the tetherin expression plasmids were serially diluted from 200 ng to 12.5 ng per transfection and pCR3.1 was used as a DNA filler. Stable WT and mutant hu-tetherin-HA expressing 293T-derived cell lines were generated by retroviral transduction, as previously described [8].

### Virion yield assays

Transfected 293T cells were place in fresh medium at 20 hrs post transfection and virion containing cell supernatants were harvested and filtered (0.2 µm) at 40 hrs post transfection. Infectious virus release was determined by inoculating, in triplicate, sub-confluent monolayers of HeLa-TZM cells seeded in 48 well plates at 2.5×10^4^ cells/well with 50 µl of serially diluted supernatants. At 48 hrs post infection, ß-galactosidase activity was determined using GalactoStar reagent as per the manufacturer's instructions. The remainder of the virion containing supernatant (450 µl) was layered onto 800 µl of 20% sucrose in PBS and centrifuged at 20,000 *g* for 90 minutes at 4°C and virion yield determined by western blot assays

### Western Blot assays

Pelleted virions and the corresponding cell lysates were resuspended in SDS-PAGE loading buffer and separated on NuPAGE Novex 4–12% Bis-Tris Mini Gels (Invitrogen). Proteins were blotted onto nitrocellulose membranes. Thereafter, HIV-1 Gag or capsid proteins, as well as tagged tetherin proteins were revealed using anti-capsid and anti-HA antibodies and chemiluminescent detection reagents, as described previously.

### Microscopy

293T cells stably expressing either WT or mutant (delGI/T45I) tetherin-HA were plated on poly-D-lysine coated dishes (Mattek). The following day the cells we infected with VSV-G pseudotyped HIV-1(WT) or HIV-1(delVpu) variants that carried Cerulean-FP (CFP) embedded in the stalk region of the matrix domain of Gag. The virus dose was chosen so that approximately 40% of the cells were infected. At 48 h after infection, cells were fixed but not permeabilized in order to confine tetherin-HA staining to surface expressed protein. Fixed cells were sequentially incubated with an anti-HA monoclonal antibody (Covance) followed by an anti mouse IgG Alexafluor 594 conjugate. The cells were imaged using a Deltavision microscopy suite and infected cells (identified by the presence of CFP fluorescence) were scored for the presence of intense tetherin-HA staining on the cell surface.

### Tetherin sequence retrieval and analysis

The aforementioned human, chimpanzee, rhesus monkey, African green monkey and mouse sequences were included in an analysis for positive selection. In addition, tetherin genes were amplified from lymphocyte RNA from several rhesus macaques (n = 8) pigtail macaques (n = 1), crab eating macaques (n = 6) and sooty mangabeys (n = 6). These were cloned using a kit TOPO-TA kit (Invitrogen) and the sequences of multiple clones determined. Representative alleles were included in the analysis described below. Additional tetherin sequences from gorilla, gibbon, and marmoset were retrieved from the raw data archives of ongoing genome sequencing projects by TraceBLAST (http://www.ncbi.nlm.nih.gov/BLAST/Blast.cgi), using each of the hu-tetherin coding exons as a separate query. Sequences were aligned using Macvector, and adjusted manually. Codon-based nucleotide alignments were used in conjunction with phylogenetic trees generated using the DNAPARS program (PHYLIP) as input for the random effects likelihood (REL) program (HyPhy) to detect positive selection. Input files for analysis using CODEML in the PAML suite (version 3.14) were generated by first aligning amino-acid sequences using the CLUSTAL-W algorithm, converting the alignment back to nucleotides, and adjusting manually where necessary using MEGALIGN (DNASTAR, Madison, WI). Tree files were generated by Neighbor-Joining, and sites with dN/dS>1 were identified using the resulting tree or a tree constrained to accept the known major branches of primate evolution as input, with similar results. The F3X4 model of codon frequencies was used for all analyses in CODEML. Paired, nested models of sequence evolution implemented in CODEML (M0, M3; M1, M2; M7, M8) were also compared using the likelihood ratio test. Evaluation with the chi-square test assumed either 4 degrees of freedom (M0, M3) or 2 degrees of freedom (M1,M2; M7, M8).

## Supporting Information

Figure S1 Western blot analysis of tetherin-HA and HA-tetherin expression. In each case cells were transfected with 50 ng of a tetherin expression plasmid. (A) Intact primate and mouse tetherin-HA or HA-tetherin proteins, as indicated, see Fig. 1. (B) Chimeric tetherin-HA proteins, in which the TM domans were exchanged, see Fig. 2. (C) Individual mutant hu-tetherin-HA proteins, see Fig. 3, 4. (D) Combination mutant hu-tetherin-HA proteins, see Fig. 5.
(1.19 MB PDF)

Figure S2 Alignment of tetherin protein sequences, from human (hu), chimpanzee (Cpz), gorilla (Gor), gibbon (Gib), African Green monkey (Agm), macaques (*nemenstrina*, Mac(n)1,2), (*mulatta*, Mac(m)db,1,2,3), *fasicularis*, Mac(f)1,2), sooty mangabeys (Sm1,2) and Marmoset (Mar). Alignments were derived using Macvector, with adjustments by hand. Corresponding codon-matched nucleotide alignments were used in analyses of positive selection (see Fig. 7).
(0.17 MB PDF)
