# Supplementary figures and images for: Species-Specific Activity of HIV-1 Vpu and Positive Selection of Tetherin Transmembrane Domain Variants
Source: PLoS Pathog. 2009 Feb 13;5(2):e1000300. doi: 10.1371/journal.ppat.1000300 (PMC2633611; doi:10.1371/journal.ppat.1000300)

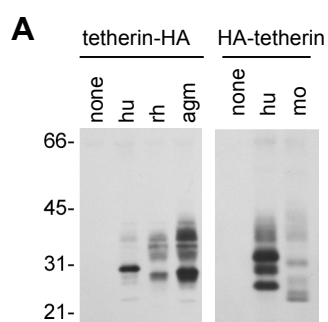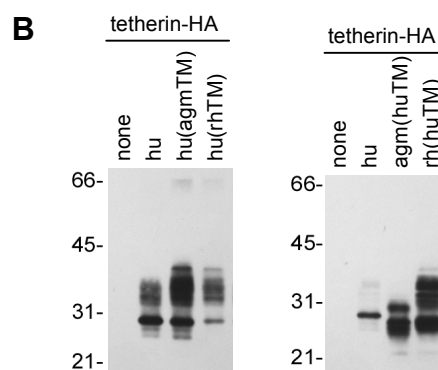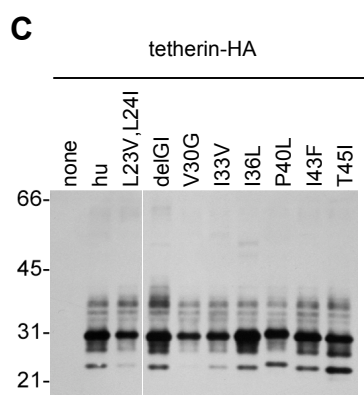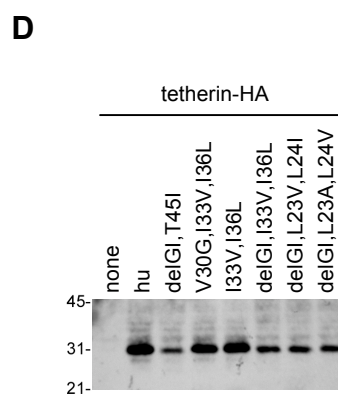

Supplement: Figure S1 — Western blot analysis of tetherin-HA and HA-tetherin expression. In each case cells were transfected with 50 ng of a tetherin expression plasmid. (A) Intact primate and mouse tetherin-HA or HA-tetherin proteins, as indicated, see Fig. 1. (B) Chimeric tetherin-HA proteins, in which the TM domans were exchanged, see Fig. 2. (C) Individual mutant hu-tetherin-HA proteins, see Fig. 3, 4. (D) Combination mutant hu-tetherin-HA proteins, see Fig. 5. (1.19 MB PDF) [file ppat.1000300.s001.pdf]
