# Supplementary material for: Species-Specific Activity of HIV-1 Vpu and Positive Selection of Tetherin Transmembrane Domain Variants
Source: PLoS Pathog. 2009 Feb 13;5(2):e1000300. doi: 10.1371/journal.ppat.1000300 (PMC2633611; doi:10.1371/journal.ppat.1000300)
Supplement: Figure S2 — Alignment of tetherin protein sequences, from human (hu), chimpanzee (Cpz), gorilla (Gor), gibbon (Gib), African Green monkey (Agm), macaques (nemenstrina, Mac(n)1,2), (mulatta, Mac(m)db,1,2,3), fasicularis, Mac(f)1,2), sooty mangabeys (Sm1,2) and Marmoset (Mar). Alignments were derived using Macvector, with adjustments by hand. Corresponding codon-matched nucleotide alignments were used in analyses of positive selection (see Fig. 7). (0.17 MB PDF) [file ppat.1000300.s002.pdf]

```
                    10        20        30        40        50        60
Hu        MASTSYDYCRVPM-----EDGDKRCKLLLGIGILVLLIIVI--LGVPLIIFTIKANSEACRDGLR
Cpz       MASTLYDYCRVPMDDIWKKDGDKRCKLLLGIGILMLLIIVI--LGVPLIIFTIKANSEACRDGLR
Gor       MASTLYDYCRVPMDAILKKDGDKRCKLLLGIGILVLLIIVI--LGVPLIIFTIKANSEACRDGLR
Gib       MAPTLYDYCRVPMDDIWKKDGDKRCKLLLGIGILVLLIIVS--LGVPLIIFTIKANSEACRDGLR
Agm       MAPILYDYCKMPMDDICKEDRDKCCKLA--VGILGLLVIVL--LGVPLIFFIIKANSEACQDGLR
Mac(n)1   MAPILYDYCKMPMDDIWKEDGDKRCKLV--VGILGLLVIVL--LGVLLIFFIIKANSEACQDGLR
Mac(n)2   MAPILYDYCKRPMDDIWKEDGDKRCKLV--VGILGLLVIVL--LGVLLIFFIIKANSEACQDGLR
Mac(m)db  MAPILYDYRKMPMDDIWKEDGDKRCKLV--VGILGLLVIVL--LGVPLIFFIIKANSEACQDGLR
Mac(m)1   MAPILYDYCKMPMDDIWKEDGDKRCKLV--IGILGLLVIVL--LGVLLIFFIIKANSEACQDGLR
Mac(m)2   MAPILYDYCKMPMGDIWKEDGDKRCKLV--IGILGLLVIVL--LGVLLIFFIIKANSEACQDGLR
Mac(m)3   MAPILYDYCKMPMGDIWKEDGDKRCKLV--IGILGLLVIVL--LGVLLIFFIIKANSEACQDGLR
Mac(f)1   MAPILYDYCKMPMGDIWKEDGDKRCKLV--VGILGLLVIVL--LGVLLIFFIIKANSEACQDGLR
Mac(f)2   MAPILYDYCQMPMGDIWKEDGDKRCILV--VGILGLLVIVL--LGVLLIFFIIKANSEACQDGLR
Sm1       MAPILYDYCRMPMGDIWKEDGDKRCKLV--VGILGLLVVVL--LGVPLIFFIIKANSEACQDGLR
Sm2       MAPILYDYCRMPMGDIWKEDGDKRCKLV--VGILGLLVIVL--LGVPLIFFIIKANSEACQDGLR
Mar       MAPTLFHYSLVPMDDFLKHESKERCKLPVGTGVVVLLVIVAVALGVLLIVYVKKANSEACQEGHR

              70        80        90        100       110       120       130
Hu        AVMECRNVTHLLQQELTEAQKGFQDVEAQAATCNHTVMALMASLDAEKAQGQK---KVEELEGEI
Cpz       AVMECRNVTHLLQQELTEAQKGFQDVEAQAATCNHTVMALMASLDAEKAQGQK---KVEELEEEI
Gor       AVMECRNVTHLLQQELTEAQKGFQDVEAQAATCNHTVMALMASLDAEKAQGQK---KVEELEGEI
Gib       AVMECRNVTHLLQQELTEAQKGFQDAEAQAATCNQTVMALMASLDAEKAQGQK---KVEELEGEI
Agm       AVMECHNVTYLLQQELAEAQRGFRDAEAQAVTCNQTVMALMASLDAEKAQGRK---KVEELEGEI
Mac(n)1   AVMECRNVTYLLQQELAEAQRGFRDAEAQAVTCNQTVMALMASLDAEKAQGRK---KVEELEGEI
Mac(n)2   AVMECRNVTYLLQQELAEAQRGFRDAEAQAVTCNQTVMALMASLDAEKAQGRK---KVEELEGEI
Mac(m)db  AVMECRNVTYLLQQELAEAQRGFRDAEAQAVTCNQTVMALMASLDAEKAHGRK---KVEELEGEI
Mac(m)1   AVMECRNVTYLLQQELAEAQRGFRDAEAQAVTCNQTVMALMASLDAEKAQGRK---KVEELEGEI
Mac(m)2   AVMECRNVTYLLQQELAEAQRGFRDAEAQAVTCNQTVMALMASLDAEKAQGRK---KVEELEGEI
Mac(m)3   AVMECRNVTYLLQQELAEAQRGFRDAEAQAVTCNQTVMALMASLDAEKAHGRK---KVEELEGEI
Mac(f)1   AVMECRNVTYLLQQELAEAQRGFRDAEAQAVTCNQTVMALMASLDAEKAQGRK---KVEELEGEI
Mac(f)2   AVMECRNVTYLLQQELAEAQRGFRDAEAQAVTCNQTVMALMASLDAEKAQGRK---KVEELEGEI
Sm1       AVMECRNVTYLLQQELAEAQRGFRDAEEQAVTCNQTVVALMASLDAEKAQGRK---KVEELEGEI
Sm2       AVMECRNVTYLLQQELAEAQRGFRDAEAQAVTCNQTVVALMASLDAEKAQGRK---KVEELEGEI
Mar       AVMECRNVTHLLEQELTRAQDGFRDAEAQIATYNQTVMTLMASLDKEKAHSLDAEKKVEELEGEI

                   140       150       160       170       180       190
Hu        TTLNHKLQDASAEVERLRRENQVLSVRIADKKYYPSSQDS-SSAAAPQLLIVLLGLSALLQ
Cpz       TTLNHKLQDASAEVERLRRENQVLSVRIADKKYYSSSQDS-SSAAAPQLLIVLLGLSALLQ
Gor       TTLNHKLQDASAEVERLRRENQVLSVRIADKKYYPSSQDS-SSAAAPQLLFVLLGLSALLQ
Gib       TTLNHKLQDASAEVERLRRENQVLSVRIADKKYSASSQDS-SSAAAPQILILLLGLSALL
Agm       TTLNHKLQDASAEVERLRRENQVLNVRIADTNS-ASSQDS-SCAAEPPLLILLLGLGALLL
Mac(n)1   TTLNHKLQDASAEVERLRRENHVLNARIADTDS-ASSQDS-SCAAEPPLLILLLGLSALLL
Mac(n)2   TTLNHKLQDASAEVERLRRENHVLNARIADTDS-ASSQDS-SCAAEPPLLILLLGLSALLL
Mac(m)db  TTLNHKLQDASAEVERLRRENHVLNARIADTDS-ASPQDS-SCAAEPPLLILLLGLSALLL
Mac(m)1   TTLNHKLQDASAEVERLRRENHVLNARIADTDS-ASPQDS-SCAAEPPLLILLLGLSALLL
Mac(m)2   TTLNHKLQDASAEVERLRRENHVLNARIADTDS-ASPQDS-SCAAEPPLLILLLGLSALLL
Mac(m)3   TTLNHKLQDASAEVERLRRENHVLNARIADTDS-ASSQDS-SCAAEPPLLILLLGLSALLL
Mac(f)1   TTLNHKLQDASAEVERLRRENHVLNARIADTDS-ASSQDS-SCAAEPPLLILLLGLSALLL
Mac(f)2   TTLNHKLQDASAEVERLRRENHVLNARIADTDS-ASSQDS-SCAAEPPLLILLLGLSALLL
Sm1       TTLNHKLQDASAEVERLRRENHVLNARIADTDS-ASSQDS-SCAAEPPLLILLLGLGALLL
Sm2       TTLNHKLQDASAEVERLRRENHVLNARIADTDS-ASSQDS-SCAAEPPLLILLLGLSALLL
Mar       TTLNQKLQDASMEVERLRRKNQDLSARIADQKNSASSLKDTSSAAAPLLLILLLGLSARLL
```
